# The regulation of pedicle initiation by androgens in sika deer (Cervus nippon)

**DOI:** 10.3389/fcell.2025.1708732

**Published:** 2026-03-31

**Authors:** Zhen Liu, Xiongzhi Li, Qianqian Guo, Guangyu Li, Hongmei Sun, Jiangbin Zou, Baorui Xing, Rana Waseem Akhtar, Yingyan Guo, Haiping Zhao

**Affiliations:** 1 College of Animal Science and Technology, Qingdao Agricultural University, Qingdao, China; 2 Shandong Engineering Research Center of Livestock Products Functional Utilization, Shandong, China; 3 Institute of Antler Science and Product Technology, Changchun Sci-Tech University, Jilin, China; 4 Institute of Special Products, Chinese Academy of Agricultural Sciences, Changchun, China; 5 Department of Pharmacy, Peking University, Shenzhen, China; 6 Department of Animal Breeding and Genetics, Faculty of Veterinay and Animal Sciences, The Islamia University Bahawalpur, Bahawalpur, Pakistan; 7 Chifeng Vocational College of Applied Technology, Chifeng, China

**Keywords:** androgen, antler generation, antlerogenic periosteum, pedicle initiation, proteomics

## Abstract

**Background:**

Deer antlers have the potential to contribute to a range of novel biomedical models. Although androgen is undoubtedly an undisputed prerequisite for pedicle initiation (initial stage of antler formation), the molecular mechanisms underlying androgen stimulation remain unclear.

**Methods:**

Four juvenile male deer (Cervus nippon) were selected: one intact individual and three castrated ones. The castrates were further divided to receive either testosterone or vehicle injections. The antlerogenic periosteum (AP) tissues overlying each frontal crest were collected at successive time points for subsequent analyses. Proteomic analysis was performed using two-dimensional difference gel electrophoresis (2D-DIGE), while cell proliferation and differentiation were assessed through *ex vivo* tissue explant cultures, *in vitro* osteoblast induction, and transcriptomic analyses.

**Results:**

Exogenous androgen induced pedicle initiation. Among the differentially-expressed proteins (DEPs) identified, ACTB, VIM, p53, RNASEL, and CALR were notable for their direct interactions with the androgen receptor (AR). Pathways related to the cell cycle, gene expression, protein metabolism, and signal transduction were found to play key roles in activating pedicle initiation. In serum-free medium, testosterone promoted the proliferation of AP cells in *ex vivo* tissue explant cultures; however, it showed no effect on osteogenic differentiation of these cells.

**Conclusion:**

Our findings indicate that testosterone serves as the key factor triggering pedicle initiation. However, this effect likely occurs through the promotion of AP cell proliferation rather than their osteogenic differentiation. Furthermore, the results suggest that CALR may play a pivotal role in regulating the transcription of AR downstream genes during pedicle initiation. These results provide new insights into androgen-regulated mechanisms in deer pedicle initiation.

## 1 Background

In most cases, androgens serve as the primary regulators of the development, maintenance and enhancement of male secondary sexual characters, including the prostate glands, masculine muscle, body size, androgen-sensitive hair follicles and deer antlers (MacLean et al., 1993; Mooradian et al., 1987). Since secondary sexual characters are often elaborate and exaggerated, it is likely that they are costly to produce and maintain (Møller, 1996). Nevertheless, a tremendous antler tissue mass (up to 30 kg) (Darwin et al., 1981; Li et al., 2009; Suttie et al., 1995), with a growth rates reaching up to 2.7 cm a day (GOSS and Richard, 1985), can form annually from each pedicle stump (a permanent protuberance that sustains annual antler regeneration) of male deer (Richard and Goss, 1984), which is unimaginable to expend tremendous amounts of energy to develop exaggerated sexual traits in nature. Antlers are commonly regarded as weapons used by deer in intraspecific combat, yet few recognize their remarkable and unique biological characteristics, features that hold great potential for contributing to the development of diverse novel biomedical models (Goss and Powel, 1985; Li and Suttie, 2012; Li and Suttie, 2001; Li et al., 2014). The atypical features of antler “stem” cells, such as expression of key embryonic stem cell markers and pluripotency, serve as an invaluable model for understanding how some of the attributes of embryonic stem cells can be retained in postnatal tissues (Li et al., 2009; Li et al., 2014). Most studies suggest that androgens have inhibitory effects on stem cell functions, and either anti-androgens or a testosterone depletion could negate these effects (Chang et al., 2006; Leskelä et al., 2006). Nevertheless, androgens could upregulate androgen receptor expression to stimulate osteoblast proliferation and differentiation (Kasperk et al., 1997; Kasperk et al., 1989). Handelsman (Handelsman, 2006) reported that androgen action is more complex than any other steroids. Taken together, studies on antler development under androgen regulation provide valuable insights for research on secondary sexual characteristics, regenerative medicine, and developmental biology.

Antler development can generally be classified into two main categories: antler generation, which includes pedicle and first antler formation, and antler regeneration, referring to the annual renewal of antlers from the pedicles (Li and Suttie, 2001; Li et al., 2009; Li et al., 2014). The unique attributes of antler generation and regeneration result from the developmental origin, as direct derivatives of antlerogenic periosteum (AP), a tissue that overlies each frontal crest in prepubertal deer (Li et al., 2009; Li et al., 2014; Suttie et al., 1995). Suttie et al. (1984). reported that pedicle initiation is associated with rising and elevated plasma testosterone levels as deer approach puberty. The close association between the initial development of AP and rising androgen levels has been functionally confirmed through experimental studies. Castration of prepubertal male deer results in the failure of antler generation. The abnormality of castration can be overcome by administration of exogenous testosterone (Jaczewski, 1982; Li et al., 2003; Li et al., 2009). Although the AP tissue overlies each frontal crest including male and female deer, but latter (except for reindeer) are unable to develop pedicles and first antlers, unless being treated with exogenous androgen hormones (Li et al., 2003). Clearly, the initial development of AP tissues is closely linked to circulating androgen concentrations, with elevated androgen levels being essential for pedicle initiation. Surprisingly, despite all this full dependency of pedicle formation on androgen hormones *in vivo*, the *in vitro* studies failed to demonstrate any direct mitogenic effects of testosterone or dihydrotestosterone on the AP cells within a very wide range of doses including physiological level (Li et al., 1999). In addtion, there is little published information on the molecular mechanisms of androgen regulation in the initial development of AP tissues.

In the present study, two-dimensional difference gel electrophoresis (2D-DIGE) was employed to identify the differentially-expressed proteins (DEPs) between the two contrasting tissue states (the androgen-activated AP tissue and the undeveloped AP tissue). Analyzed the DEPs profiles of pedicle initiation. The DEPs associated with pedicle initiation were analyzed, and the effects of testosterone on AP cell proliferation and osteogenic differentiation were further investigated. This research aimed to identify key regulatory proteins and signaling pathways underlying the molecular mechanisms of androgen stimulation during pedicle initiation. The findings contribute valuable insights to the fields of proteomics, secondary sexual character development, regenerative medicine, and developmental biology.

## 2 Methods

The work has been reported in line with the ARRIVE guidelines 2.0.

### 2.1 Animals

Four 10-month-old sika deer (Cervus nippon) stag calves (52–58 kg) were selected prior to pedicle development for AP tissue sampling and androgen treatment: DI, deer intact (no treatment, just grouped together with the treated deer); DC, deer for control; DT, deer treated with testosterone undecanoate (250 mg/deer); and DS, deer treated with the solvent of testosterone undecanoate (tea oil). The experimental deer were maintained in captivity from the start to the completion of the study. All the experiments were performed in accordance with the guideline and study protocols of the Qingdao Agricultural University. All deer were housed at Haiyang Guangxing Agricultural Technology Co., Ltd. Following sample collection, the deer were maintained under standard breeding conditions.

### 2.2 Treatment and sampling

The stag calves (DC, DT and DS) were castrated on 7 April. Castration was conducted under general anesthesia using Xylazine Hydrochloride (Dunhua Shengda Animal Medicine Co. Ltd., China) and sedation was reversed using Luxingning (Huamu Animal Health Products Co. Ltd., Jilin Province, China). Blood sampling was carried out on the day of castration and thereafter at biweekly intervals. A 10 mL blood sample was taken each time from jugular vein into a pre-heparinised tube and immediately centrifuged to collect plasma. The plasma was frozen at −20 °C for later hormone assay.

**FIGURE 1:**
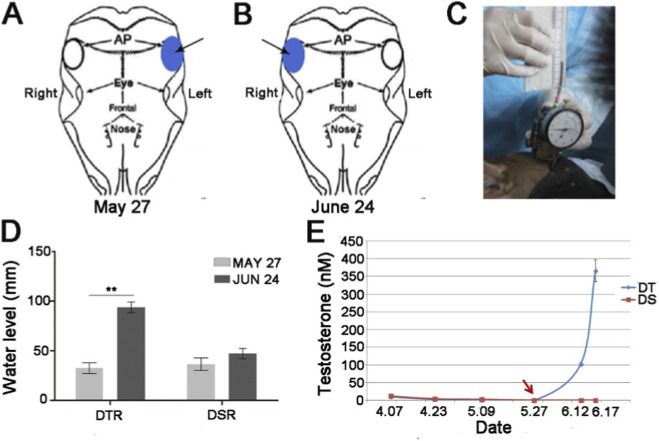
**(A)** The left side of AP tissue (arrow). **(B)** The right side of AP tissue (arrow). **(C)** Detection of pedicle growth using a pedicle growth detector. **(D)** Changes in water level (DTR and DSR). **(E)** Changes in concentration of testosterone (DT and DS). Date of injection of Testosterone and tea oil (red arrow).

AP tissue from the left side (Figure 1A) of each deer was taken at the time when pedicle development of DI had convincingly initiated (May 27), but no signs of pedicle growth were detected in the rest of deer (Li et al., 1994). These sampled left-side AP tissues were assigned DCL, DTL and DSL respectively. DT was injected with 2 mL (250 mg) testosterone undecanoate (Xianju Pharma, CN) in solvent (tea oil) i. m. At the neck; and the DS injected with 2 mL tea oil. Each piece of collected AP tissue was divided into 3 parts: one (further divided into 3 parts randomly) was stored in liquid nitrogen for protein extraction, one stored in RNA Stabilization Solution (AM7020, Thermo), and one stored in 4% Paraformaldehyde (P1110, solarbio) for histology and immunohistochemical (IHC) analysis.

Initiation status of the pedicle growth was detected biweekly after castration and injection of T or tea oil using our pedicle growth detector (Figure 1C) (Li et al., 1994). AP tissues from the right side (Figure 1B) of DT and DS were taken after pedicle growth of DT had convincingly initiated (June 24) using the detector. These sampled right-side AP tissues were designated DTR and DSR respectively. AP on the right side of DC was missing due to the deer was accidently died before the time of AP sampling. The collected AP tissues were treated as the before mentioned procedure. Collectively, 15 pieces of AP tissues (9 from left- and 6 from right-side AP) were obtained for analysis using 2D-DIGE.

### 2.3 2D-DIGE

Reagents and equipment were bought from GE Healthcare unless otherwise noted.

#### 2.3.1 Protein extraction and preparation

Each piece of frozen AP was pre-cooled with liquid nitrogen and with a continuous supply of liquid nitrogen during grinding until the fine powder was achieved. The powder was re-suspended in 2 mL of lysis buffer (7 M Urea, 2 M Thiourea, 4% 3-[(3-Cholamidopropyl)dimethyl-ammonio]-1-propane sulfonate (CHAPS), 1% protease inhibitor cocktail, and 200 U/mL Benzonase), and incubated for 1 h at room temperature. Supernatants were collected after centrifugation at 15,000 *g* for 15 min at 4 °C. Duo to the presence of high-abundant proteins, especially albumin and IgG, a multi-step-sample-preparation-procedure was applied before carrying out 2D-DIGE (Supplementary Figure S1). Albumin/IgG was removed using Albumin/IgG Removal Kit (89,875, Thermo) according to the manufacture’s protocol. The proteins were then precipitated and re-dissolved in 30 mM Tris-HCl, PH 8.8, 7 M Urea, 2 M Thiourea, 4% CHAPS using the 2-D Clean-up Kit, which helps to remove interfering substances. If not used immediately, the protein solutions were frozen at −80 °C after addition of protease inhibitor cocktail. The concentrations of protein solution were measured using the RC DC Protein Assay Kit (5,000,119, Bio-Rad).

For protein labeling with CyDye DIGE Fluor, 50 µg protein from each AP sample was labeled with a ratio of 400 pmol CyDye using a minimal labeling kit. An internal pooled standard (IPS, 50 μg, labeled with Cy2) comprising equal amount of each protein sample was run along with a pair of samples (50 µg per sample) labeled with Cy3 and Cy5 on each gel (Table 1). In total, eight gels were run in the experiment with a total of 150 µg mixed labeled protein each. After labeling, the three labeled samples for each gel were pooled, an equal volume of 2X sample buffer was added, and rehydration solution was added to make a total volume of 450 µL. After vortexing, the 450 µL pooled sample for each gel was left 15 min on ice in the dark.

**TABLE 1:** Fluorescent labeling for samples in each gel.

Gel NO.	Cy2	Cy3	Cy5
Gel 1	IPS	DCL1	DTR1
Gel 2	IPS	DCL2	DSL1
Gel 3	IPS	DTR2	DCL3
Gel 4	IPS	DTR3	DTL1
Gel 5	IPS	DSL2	DTL2
Gel 6	IPS	DTL3	DSR1
Gel 7	IPS	DSL3	DSR2
Gel 8	IPS	DSR3	​

IPS, internal pooled standard; DCL, the AP, on the left side of DC; DTL, the AP, on the left side of DT; DTR, the AP, on the right side of DT; DSL, the AP, on the left side of DS; DSR, the AP, on the right side of DS.

#### 2.3.2 Isoelectric focusing and gel electrophoresis

Immobiline Dry Strip, PH 3-10 NL, 24 cm gels were rehydrated overnight in 450 µL pooled samples in an IPG box. Isoelectric focusing (IEF) was carried out using an Ettan IPG phor 3 and the following procedures: 12 h at 50 V, 0.5 h at 200 V, 1.5 h at 600 V (gradient), 2.5 h at 1000 V (gradient), 3 h at 6000 V (gradient), 1.5 h at 9000 V (gradient), 3.5 h at 9000 V. Strips were equilibrated immediately for 15 min in 6 M Urea, 2% SDS, 0.375 M Tris-HCl PH 8.8, 20% glycerol, containing 1% DTT, and then for 15 min in the same buffer containing 2.5% iodoacetamide. Strips were transferred onto 12% SDS polyacrylamide gels, which had been pre-casted in low fluorescence glass plates, after washing with ultrapure water, and covered by 0.5% melted agarose and traces of bromophenol blue. The second dimension separation was then carried out at 3 W/gel for 4.5 h using Ettan DALT six electrophoresis unit.

#### 2.3.3 Differential image analysis

The electrophoretic gels were scanned using a Typhoon FLA 9500 at excitation/emission wavelengths of 488/520 (40), 532/580 (30) and 633/670 (30) for Cy2, Cy3 and Cy5 respectively. Scanning pixel size was 100 microns. The three gel images (protein spot maps) and statistical analysis were done using the DeCyder 2D Differential Analysis Software (V7.2) by Batch Processor, Image Loader, Differential In-gel Analysis (DIA), Biological Variation Analysis (BVA) and Extended Data Analysis (EDA). Firstly, protein spots were automatically matched and detected in different gels. The estimated number of spots was set to 2,500, spot slope and volume were set to 1.12 and 20,000 respectively. The insufficiently reproducible spots were excluded and the quantification was done only on the matched spots. Protein spots with changes in abundance ratio >1.5-fold, *p* ≤ 0.05 were assigned on the basis of t-test and one-way ANOVA analysis to a pick list for following MALDI-TOF/TOF MS/MS analysis. Briefly, 3 comparisons were performed: 1, All DEPs were obtained from the comparisons between DTR (androgen-activated) and DCL, DTL and DSL (androgen-unactivated) using t-test analysis; 2, DEPs obtained from the comparisons between DCL, DTL and DSL (all are the androgen-unactivated AP tissues) using one-way ANOVA analysis; 3, DEPs obtained from the comparisons between DSR and DCL, DTL and DSL (sampled on 27 May) using t-test analysis. The finally-selected-DEPs for further identification were obtained through the following equation: DEPs from Comparison 1 minus those from Comparisons 2 (eliminating individual difference) and 3 (eliminating time difference). Selection of each protein spot for clustering analysis using EDA was based on the following criteria: a protein spot must be present on at least 19 gels (account for 75% of total gels). After 2D-DIGE imaging and statistical analysis, the gels were subjected to silver staining for spot visualization and manual picking. Peptide mass spectrometric analysis was performed using 5800 MALDI-TOF/TOF/MS (AB SCIEX). Protein identification was conducted using the Mascot 2.2 software.

### 2.4 Bioinformatics analysis

Enrichment analysis of Gene Ontology (GO) of the identified DEPs was performed in default setting using DAVID 6.8. To gain more insight into the possible biological functions of androgen, pathway analysis was performed using KOBAS 3.0 which allows us to identify the enriched pathways (statistically significantly) from multiple commonly used databases including the Encyclopedia of Genes and Genomes (KEGG) pathway, Reactome pathway, BioCyc pathway and Protein Analysis Through Evolutionary Relationships (PANTHER) pathway databases (Kasperk et al., 1997; Xie et al., 2011). The following statistical test methods were used: hypergeometric test and Fisher’s exact test with Benjamini and Hochberg FDR correction and only the terms that were with corrected *p*-value <0.01 were shown. Protein-protein interaction analysis between androgen receptor (AR, UniProtKB-P10275) and the identified DEPs was performed using String (Version 10.5) to identify the proteins that interact with AR directly or indirectly.

### 2.5 Validation of 2D-DIGE results using real-time quantitative polymerase chain reaction (qPCR)

Total RNA from all the AP tissues were extracted using Trizol (15,596,026, Invitrogen) according to the manufacturer’s protocol. The reverse transcription was performed using PrimeScript™ II 1st Strand cDNA Synthesis Kit (6210A, Takara) according to manufacturer’s instructions. Gene-specific primers (Table 2) were designed using Primer Premier 6.0 (Premier Biosoft, United States). qPCR was performed using a Roche LightCycler 480 with FastStart Universal SYBR Green Master (Rox) (4,913,850,001, Roche) with the following procedures: 10 min at 95 °C (pre-incubation), 10 s at 95 °C, 30 s at 61 °C, 15 s at 72 °C (amplification, 45 cycles), and 5 s at 95 °C, 1 min at 65 °C, 97 °C continuously (melting curve), followed by quantitative analysis using the Roche LightCycler 480 software. GAPDH was used as the house-keeping gene for normalization. The data were analyzed using the 2^−ΔΔCq^ method and the results were presented as mean ± SD. Student’s t-test was used to evaluate statistical significance using GraphPad Prism 5 (version 5.01, United States). All reactions were performed in three technical repeats.

**TABLE 2:** Primers used for Q-PCR.

Gene name	Forward (5′-3′)	Reverse (5′-3′)
*P53*	TAT​TTG​GAC​GAC​CGG​AAC​ACT	TCC​CAG​CAG​GTT​ACC​ACG​A
*MCM10*	AGA​ACG​GCT​GTG​TCA​GGA​TGG​T	TGG​CTC​CAG​ATG​TAG​GCA​AGT​CC
*SMC4*	AAG​ATG​GCA​GTG​TGG​GCA​AAG​AAA	TTG​CTT​CCA​CCG​CCA​GTC​ATT​G
*CALR*	CCT​GAT​GCC​GAT​AAG​CCT​GAA​GAC	AGG​GTT​GTC​AAT​CTC​TGG​GTG​GAT
*SRCIN1*	TGT​GGA​GGT​GGA​GGC​AGT​GAA​G	AAG​TCC​GTC​TCG​GCT​GTC​ATC​TT
*GAPDH*	ATG​TTT​GTG​ATG​GGC​GTG​AAC	CCA​GTA​GAA​GCA​GGG​ATG​ATG​TT

### 2.6 Validation of 2D-DIGE results using Western blot

Antibodies used in the Western blot analysis included: anti-GAPDH-loading control monoclonal antibody (bsm-33033M, Bioss, China), anti-SRCIN1 antibody (D153560, BBI life sciences, China), anti-Calreticulin polyclonal antibody (bs-5913R, Bioss, China), anti-SMC4 polyclonal antibody (bs-7728R, Bioss, China), anti-p53 protein (wt-p53) polyclonal antibody (bs-0033R, Bioss, China). GAPDH was used as the house-keeping protein for normalization. The expression levels of selected proteins were evaluated using ImageJ 1.48 software (National Institutes of Health, United States). All reactions were performed in three technical repeats.

### 2.7 *Ex-vivo* tissue explant culture and cell proliferation assay

The AP was taken from the frontal crests (1-year-old male sika deer) at the time when pedicle development had convincingly initiated as previously described (Li and Suttie, 2003; Li et al., 1994; Sun et al., 2012). Briefly, the AP tissues were cut into small strips (around 1.5 × 5 mm) in a 10 cm Petri dish using a pair of scalpels. Three randomly selected strips were used for following preparation of frozen sections. Other strips were floated fibrous layer side up in 24-well plates (2 strips/well) (Figure 7E) containing (1) DMEM (100 U/mL penicillin +100 μg/mL streptomycin), (2) DMEM + testosterone (Sigma, United States final concentrations: 1, 10, 50 nM), (3) DMEM + IGF1 (Abcam, United Kingdom, 10 nM), (4) DMEM + IGF1 (10 nM) + testosterone (10 nM), (5) DMEM +2% FBS, (6) DMEM +2% FBS + testosterone (10 nM), (7) DMEM +2% FBS + IGF1 (10 nM), (8) DMEM +2% FBS + IGF1 (10 nM) + testosterone (10 nM). Such that the medium was only in contact with the underside of the AP strips and the fibrous layer remained constantly exposed to the air. These strips were incubated at 37 °C in a 5% CO_2_ incubator for 12 h and exposed to 50 μM EdU (added at the 10th hour) for 2 h. Briefly, the cultured AP tissue samples and freshly AP tissues (termed as New) were embedded in optimal cutting temperature (OCT) compound, and then placed in liquid nitrogen until the OCT compound froze. EdU proliferation staining was performed using the BeyoClick™ EdU Cell Proliferation Kit with Alexa Fluor 488 (Beyotime, China) and Hoechst 33,342 (nuclear stain) according to the manufacturer’s protocol. Meanwhile, immunohistochemistry was performed using the UltraSensitive TM SP (Mouse/Rabbit) IHC Kit (MX Biotechnologies, China), according to the manufacturer’s protocol. Briefly, the sections were incubated with anti-PCNA primary antibody (1:1,000, ab18197, Abcam) over night at 4 °C, washed with PBS three times and then incubated with Alexa Fluor 647-labeled Goat Anti-Rabbit IgG (H + L) (1:500, Beyotime, China) for 2 h at room temperature. The cell proliferation rate was quantified using ImageJ software.

### 2.8 Cell differentiation and transcriptomic analysis

To further explore the effects of androgens on the AP cell differentiation, the AP cells were cultured as previously described (Li and Suttie, 2003; Liu et al., 2018; Sun et al., 2012). Briefly, the AP cells were seeded at a density of 1 × 10^4^ cells/cm^2^ in 6-well plates containing the culture medium (CM, DMEM (Gibco, United States of America) + 10% FBS (Hyclone, United States)) until they reached about 70% confluence. These cells were further cultured up to 21 days in four media: (1) the CM containing differentiation medium (DM, L-Threoascorbic acid (50 μg/mL, Solarbio, China) + dexamethasone (10 nM, Solarbio, China) + Glycero 2-phosphate disodium salt (10 mM, Solarbio, China)), (2) the CM containing DM and testosterone (10nM, sigma, United States), (3) the CM (FBS was stripped by charcoal, C-) containing DM, (4) the CM (C-) containing DM and testosterone (10 nM). These media were replaced twice or thrice a week by adding all previous regents. The osteogenic ability was detected by Alizarin Red (Yuanye, China) according to the manufacturer’s recommendation. The expression levels of SP7, SPARC and BGN mRNA of the AP cells that cultured in the CM + DM + testosterone (0 or 10 nM) for 21 days were detected by qPCR. Proliferation assay of the AP cells, cultured in the CM containing DM and testosterone (0, 5, 10, 20 nM) for 24, 48, 72 and 96 h respectively, were performed using Enhanced Cell Counting Kit-8 (Beyotime, China) according to the manufacturer’s recommendation. Total RNA was extracted from the AP cells that cultured in the CM + DM + testosterone (0 and 10 nM) for 21 days using a Trizol reagent (Invitrogen, United States) according to the manufacturer’s procedure. Transcriptome assembly, annotation, differential expression and bioinformatics analysis were performed as previously described (Ba et al., 2019).

## 3 Results

### 3.1 Exogenous androgen induced (EAI) pedicle development

A pedicle growth detector and a ruler were used to determine whether EAI pedicle had begun to grow (Li et al., 1994). For DTR, water level in the plastic tubing of the detector was significantly (*p* < 0.01) changed in 1 month of time (34.6 ± 5.8 mm to 93.6 ± 6.1 mm) (Figure 1D). Whereas, for DSR, water level was not significantly (*p* > 0.05) changed (from 37.2 ± 7.1 mm to 48.2 ± 6.9 mm) (Figure 1D). Moreover, for deer DT, the concentration of testosterone in serum was increased (from 2.36 nM to 366 nM) significantly (*p* < 0.01) after treatment of exogenous testosterone (Figure 1E). However, for deer DS, the concentration of testosterone in serum was maintained at almost undetectable levels after castration (Figure 1E).

**FIGURE 2:**
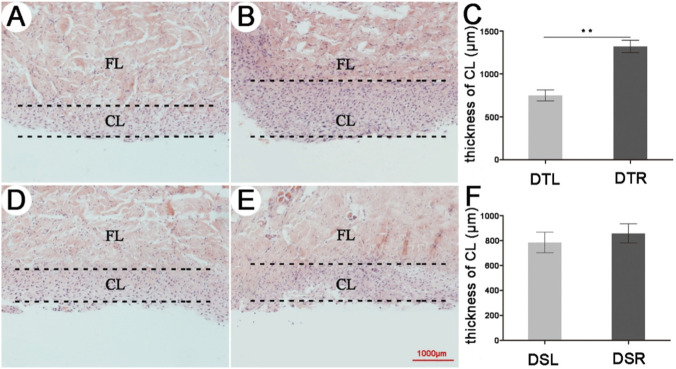
Vertical sections of the AP tissues from DTL, DTR, DSL and DSR. Hematoxylin and eosin staining. **(A)** DTL. **(B)** DTR. **(C)** Changes in thickness of the AP cellular layer of DTL and DTR. **(D)** DSL. **(E)** DSR. **(F)** Changes in thickness of the AP cellular layer of DSL and DSR. CL: cellular layer (marked by two dotted lines). FL: fibrous layer. Bar = 1,000 µm.

It is known that pedicle formation relies mainly on the AP cellular layer. Histology results showed that thickness of the AP cellular layer of DTR (1,321 ± 71 μm, n = 3) was increased significantly (*p* < 0.01) compared to that of DTL (750 ± 65 μm, n = 3) (Figure 2A–C). In contrast, thickness of the AP cellular layer between DSL (785 ± 83 µm) and DSR (857 ± 76 µm) was not significantly (*p* > 0.05) changed (Figure 2D–F).

### 3.2 DEPs identified from 2D-DIGE results

**FIGURE 3:**
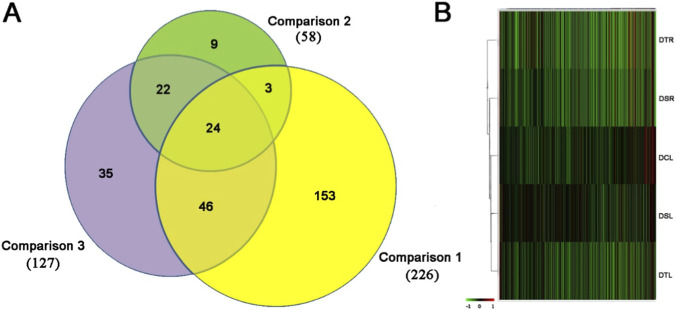
**(A)** Venn diagram showing the number of DEPs in three comparison based on the criteria: t-test (*p* ≤ 0.05) and one-way ANOVA (*p* ≤ 0.05): Comparison 1) The number of DEPs got using t-test analysis (DTR vs. DCL, DTL and DSL); Comparison 2) The number of DEPs got using one-way ANOVA analysis (DCL, DTL and DSL); Comparison 3) The number of DEPs got using t-test analysis (DSR vs. DCL, DTL and DSL). **(B)** Hierarchical cluster analysis of the AP tissue proteomes. Androgen altered the AP developmental status. Red lines represent upregulated proteins, green lines represent downregulated proteins; and black lines represent expression-level-unchanged proteins relative to the IPS.

The 2D-DIGE approach was applied to identify the proteins that possibly mediate the effects of androgen hormones on the AP cells for the initial development of pedicles. In total, 1,269 protein spots were identified and matched across all gels. Out of these protein spots, 153 (12%) (Figure 3A) were selected for following MALDI-TOF/TOF MS/MS analysis based on the results of t-test (*p* ≤ 0.05) and one-way ANOVA (*p* ≤ 0.05) analysis using BVA. The results of hierarchical cluster analysis showed that DCL, DTL, DSL and DSR were grouped together representing undeveloped AP and the DTR was the only ungrouped example representing activated AP (Figure 3B). Out of 153selected protein spots, 83 protein spots were successfully identified by mass spectrometry as the complete proteins (Supplementary Table S1).

### 3.3 Classified GO terms of the identified DEPs

**FIGURE 4:**
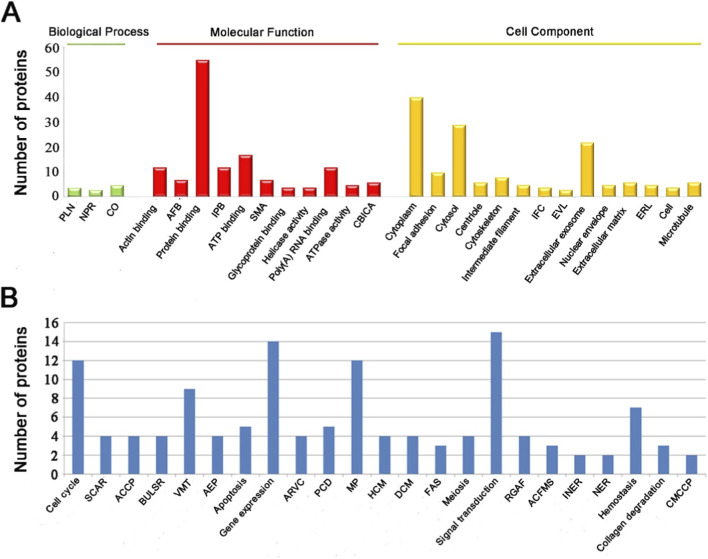
**(A)** Enriched GO terms using DAVID 6.8. Only the terms that *p* < 0.01 were shown in the identified proteins interactome. Horizontal lines and bar in different colors (green: biological process; red: molecular function; yellow: cell component) represent specific GO terms. PLN: protein localization to nucleus, NPR: neuron projection regeneration, CO: cytoskeleton organization, AFB: actin filament binding, IPB: identical protein binding, SMA: structural molecule activity, CBICA: cadherin binding involved in cell-cell adhesion, IFC: intermediate filament cytoskeleton, EVL: endocytic vesicle lumen, ERL: endoplasmic reticulum lumen. **(B)** Distribution of the pathways participated by the identified DEPs. The terms with corrected *p* < 0.01 were shown. SCAR: scavenging by class A receptors, ACCP: apoptotic cleavage of cellular proteins, BULSR: binding and uptake of ligands by scavenger receptors, VMT: vesicle-mediated transport, AEP: apoptotic execution phase, ARVC: arrhythmogenic right ventricular cardiomyopathy, PCD: programmed cell death, MP: metabolism of proteins, HCM: hypertrophic cardiomyopathy, DCM: dilated cardiomyopathy, RGAF: RHO GTPases activate formins, ACFMS: Assembly of collagen fibrils and other multimeric structures, INER: initiation of nuclear envelope reformation, CMCCP: caspase-mediated cleavage of cytoskeletal proteins.

In total, 28 GO terms (*p* < 0.01) were shown based on the results of GO analysis (Figure 4A; Supplementary Table S2). The enriched GO terms include biological process (green), molecular function (red) and cell component (yellow). The identified proteins from the biological process category were predominantly involved in protein localization to nucleus, neuron projection regeneration and cytoskeleton organization. Proteins from the molecular function group showed a significant enrichment in actin binding, actin filament bindingand protein binding. Interestingly in this category, more than half of identified proteins (55/83) were found to be involved in protein binding. Proteins from the cell component category showed a significant enrichment in cytoplasm, focal adhesion and cytosol. These may indicate that transcriptional regulation, neurogenesis and cytoskeleton organization, which affects cell division, cell motility, cell proliferation and fate (Fletcher and Mullins, 2010), played an important role during the period of pedicle initiation driven by androgen stimulation.

### 3.4 Enriched pathways for the identified DEPs

In total, 485 pathways in total for 83 DEPs were enriched based on analysis using KOBAS 3.0 (Supplementary Table S3). Out of 485 pathways, 23 pathways (corrected *p* < 0.01) were shown (Figure 4B). The most significant signaling pathways for the 83 DEPs were cell cycle (12 proteins), scavenging by class A receptors (4 proteins) and apoptotic cleavage of cellular proteins (4 proteins) etc. In addition, gene expression (14 proteins), programmed cell death (5 proteins), metabolism of proteins (12 proteins), and signal transduction (15 proteins) were also enriched that associated with pedicles initiation (Table 3). These results suggest that multiple DEPs are involved in cell cycle, gene expression and signaling transduction to promote pedicle initiation in response to androgen stimulation.

**TABLE 3:** Pathways enriched for 83 identified DEPs (androgen-activated AP vs*.* androgen-unactivated AP).

Pathways	Upregulated proteins	Downregulated proteins
Cell cycle	RAD50	SYNE2, ANKL2, CLIP1, CENPE, SYNE1, MCM10, SMC4, TOP2A, LMNB1, p53, CE290
Scavenging by class a receptors	APOE, CO1A1	APOA1, CALR
Apoptotic cleavage of cellular proteins	VIME	PLEC, LMNB1, DESP
Binding and uptake of ligands by scavenger receptors	APOE, CO1A1	APOA1, CALR
Vesicle-mediated transport	APOE, CO1A1	APOA1, CALR, ACTB, CENPE, GOGA4, MYH7B, A1AT
Gene expression	RAD50, APOE, RL3, IF4H	PRDX2, SRP54, RIOK1, MOV10, SRRM2, SYAC, ACTB, DDX21, SYHC, p53
Programmed cell death	VIME	PLEC, LMNB1, p53, DESP
Metabolism of proteins	IF4H, RL3	SRP54, LMNA, CALR, ACTB, APOA1, GMDS, IFEA, TOP2A, A1AT, p53
Signal transduction	APOE	FMNL1, MOV10, AMOL2, ACTB, VINC, GFAP, APOA1, CING, CENPE, LWA, CLIP1, p53, MIB2, PDHA1

### 3.5 2D-DIGE results confirmed using Q-PCR

**FIGURE 5:**
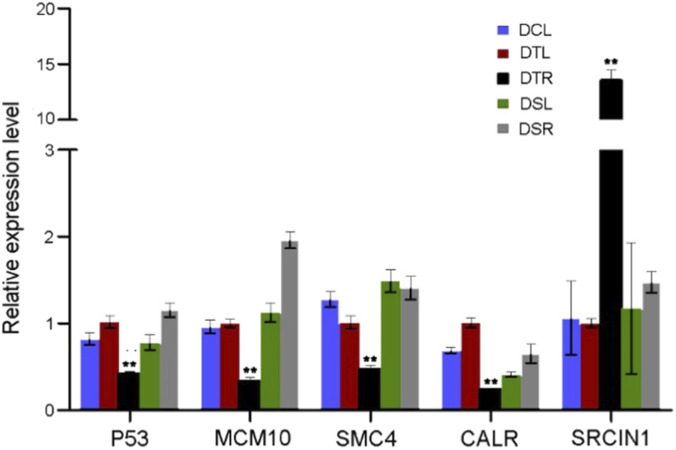
Relative mRNA expression levels of CALR, SMC4, P53, MCM10 and SRCIN1, which were validated by Q-PCR analysis normalized to GAPDH. The data are expressed as mean ± SD. **: *p* < 0.01 (the androgen-activated AP vs. the androgen-unactivated AP, n = 3).

Five proteins (CALR, SMC4, p53, MCM10 and SRCIN1) were selected from the identified DEPs based on the criteria that they either changed significantly (*p* < 0.01, |average ratio| > 2.5) or interacted with AR directly (Supplementary Figure S2) for the confirmation using Q-PCR. Their mRNA expression levels (Figure 5) were normalized using GAPDH. Compared to the undeveloped AP tissues, the relative expression level of SRCIN1 mRNA was increased significantly (*p* < 0.01), while those of CALR, SMC4, p53 and MCM10 mRNA were decreased significantly (*p* < 0.01) in the androgen-activated AP tissues (DTR). Overall, the Q-PCR results for the selected gene mRNA confirmed those of the corresponding 2D-DIGE results.

### 3.6 2D-DIGE results confirmed using Western blot analysis

**FIGURE 6:**
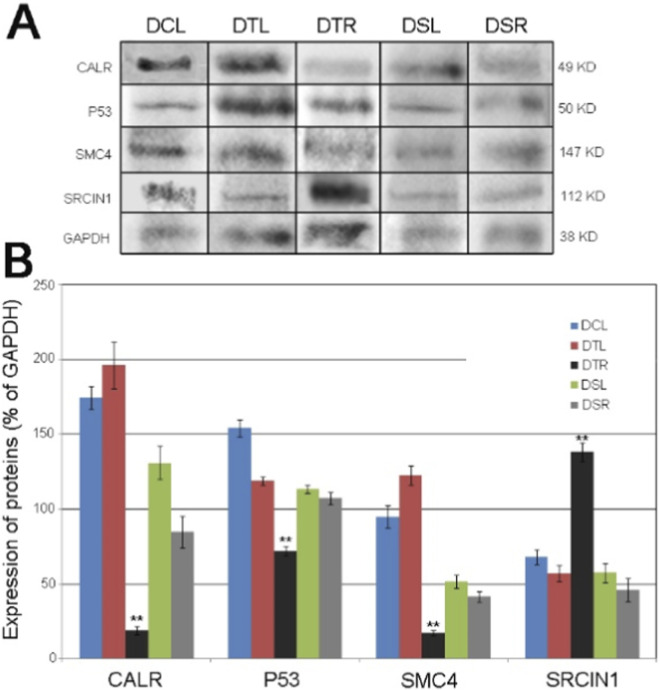
Relative protein expression levels of CALR, p53, SMC4 and SRCIN1. **(A)** western blot verification of CALR, p53, SMC4 and SRCIN1 in the different AP tissues. **(B)** Relative expression levels of CALR, p53, SMC4 and SRCIN1 in the different AP tissues.The data are expressed as mean ± SD. **: *p* < 0.01 (the developed AP vs. the undeveloped AP, n = 3).

Four proteins (CALR, p53, SMC4 and SRCIN1) were selected from the identified DEPs based on the criteria that they either changed significantly (*p* < 0.01, |average ratio| > 2.5) or interacted with AR directly (Supplementary Figure S2) for the confirmation using Western blot analysis. The results (Figure 6) showed that expression levels of CALR, p53 and SMC4 proteins in the androgen-activated AP tissues were significantly lower than those in the undeveloped AP tissues (*p* < 0.01). In addition, the expression level of SRCIN1 protein in the androgen-activated AP tissues was significantly higher than the undeveloped AP tissues (*p* < 0.01). Over all, the Western blot results were consistent with those of the corresponding 2D-DIGE. Full-length blots/gels are presented in Supplementary Figure S3,S4.

### 3.7 Cell proliferation results

**FIGURE 7:**
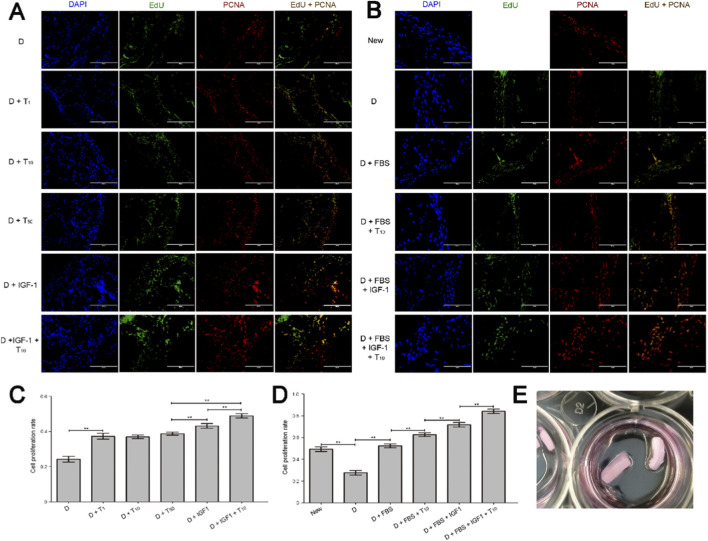
The results of cell proliferation assay through *Ex-vivo* tissue explant culture. **(A)** The EdU and PCNA staining of AP tissues cultured in serum-free medium. **(B)** The EdU and PCNA staining of AP tissues cultured in the medium containing serum. **(C)** Cell proliferation rate of the AP cells in AP tissues cultured in serum-free medium. **(D)** Cell proliferation rate of the AP cells in AP tissues cultured in the medium containing serum. **(E)** The AP tissues were cultured in 24-well plates. **(D)** DMEM, New: newly collected tissue, T_(1, 10, 50)_: testosterone concentrations: 1, 10, 50 nM.

Since the most significant signaling pathways for DEPs were mainly related to cell cycle and apoptosis, we further investigated whether testosterone could alter mitogenic effects on the AP cells that exist in the AP tissues by EdU labelling and PCNA staining (Figures 7A,B) via *Ex-vivo* tissue explant culture (Figure 7E). After all, testosterone along did not show any mitogenic effects on the AP cells *in vitro* (Li et al., 1999)*.* The results showed that, the proliferation rates of AP cells treated with different concentrations of testosterone were significantly higher than that of DMEM group, but were significantly lower than that of DMEM + IGF1 and DMEM + IGF1+ testosterone groups (*p* < 0.01; Figure 7C). Meanwhile, cell proliferation rates had not statistical difference after different concentrations of testosterone treatment (*p* > 0.05; Figure 7C). Furthermore, the AP cell proliferation rate of DMEM group was significantly lower than that of New, DMEM + FBS, DMEM + FBS + testosterone (10 nM), DMEM + FBS + IGF1 and DMEM + FBS + IGF1+ testosterone (10 nM) groups, and that of the last four groups, in order, increased significantly (*p* < 0.01; Figure 7D).

### 3.8 Cell differentiation and transcriptomic results

**FIGURE 8:**
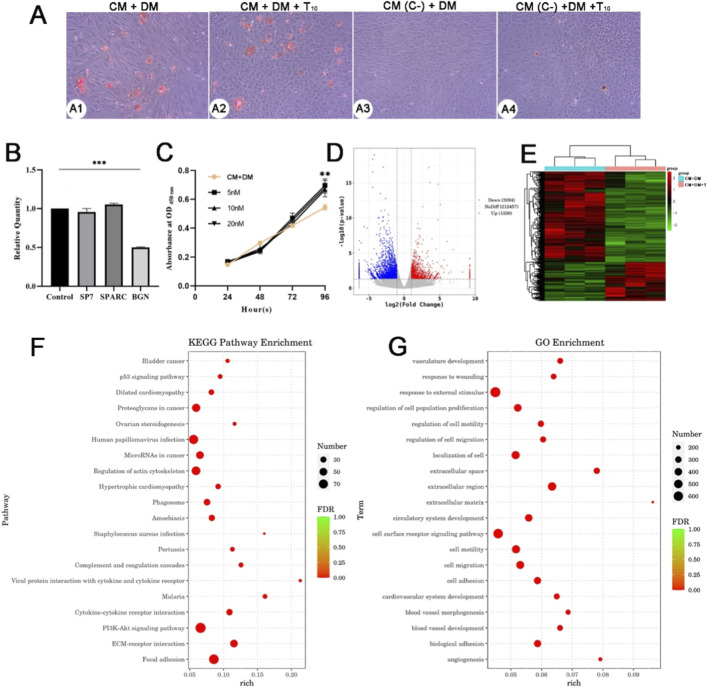
**(A)** The results of alizarin red staining. **(B)** Relative mRNA expression levels of SP7, SPARC and BGN. **: *p* < 0.01. **(C)** Results of absorbance performed using Enhanced Cell Counting Kit-8. **(D)** Volcano plot showing downregulated (blue) and upregulated (red) differentially expressed genes based on the criteria set at |log2 (fold change)| > 0.5 and FDR <0.01 as cutoff. **(E)** The results of cluster analysis of differentially expressed gene data. **(F)** KEGG enrichment analysis. **(G)** GO enrichment analysis.

To further assess the osteogenic differentiation effects of testosterone on the AP cells, induction of osteoblasts, qPCR, proliferation assay and transcriptomic analysis were performed. The results showed that there was no significant difference in the number of calcifying nodules between the CM + DM + testosterone group and CM + DM group, and the deposition of calcifying nodules were hardly observed in the CM (C-) + DM and CM (C-) + DM + testosterone groups (Figure 8A). To determine whether testosterone improved osteogenic sence of the AP cells, we examined the expression of three classic osteogenic marker genes: SP7, SPARC and BGN (Carvalho et al., 2021; Chen et al., 2019; Kram et al., 2020). The results showed that there was no significant difference in expression levels of SP7 and SPARC between the CM + DM + testosterone group and CM + DM group (*p* > 0.5; Figure 8B); nevertheless, the expression levels of BGN in the CM + DM + testosterone group were significantly lower than that in the CM + DM group (*p* < 0.01; Figure 8B). We further measured the absorbance values of CM + DM + testosterone (0, 5, 10, 20 nM) groups to determine whether testosterone elevated the survival rate of AP cells, and consequently enhanced the number of calcifying nodules. The results showed that the absorbance values of CM + DM + testosterone (5, 10, 20 nM) groups were significantly higher than that of CM + DM group (*p* < 0.01; Figure 8C).

In total, 216,871 genes were detected in both CM + DM and CM + DM + testosterone groups through RNA-seq, including 1,330 upregulated and 3,084 downregulated in the CM + DM + testosterone group based on the criteria set at |log2 (fold change)| > 2 and *p* < 0.05. The distribution status of the identified differentially expressed genes was visualized using a volcano plot (Figure 8D). The results of cluster analysis of differentially expressed gene data released that testosterone addition modified expression of some specific genes and resulted in independence between CM + DM group and CM + DM + testosterone group (Figure 8E). Remarkably, the results of KEGG and GO enrichments indicated that the most significant pathways for 4,414 differentially expressed genes were “regulation of actin cytoskeleton”, “PI3K-Akt signaling pathway”, “focal adhesion” and “p53 signaling pathway”; the most significant GO terms were “response to external stimulus”, “regulation of cell population proliferation”, “cell motility”, “localization of cell”, “cell migration” (Figures 8F,G). Taken together, these results indicate that testosterone had no effects on osteogenic ability of the AP cells, and may improve the proliferation and survival of AP cells.

## 4 Discussion

This is the first MALDI-TOF/TOF MS/MS 2D-DIGE proteomic study to examine the DEPs between androgen-activated and undeveloped AP tissues. Deer antler was used as a model to investigate the key regulated proteins that involved in pedicle initiation under the regulation of androgens. Multiple pathways (e.g., cell cycle, gene expression and signal transduction) were probably associated with pedicle initiation. CALR, SRCIN1 and p53 might be the key proteins that associated with pedicle initiation. Although testosterone had no mitogenic effects on the AP cells cultured with serum-free DMEM *in vitro* (Li et al., 1999), testosterone stimulated the proliferation of AP cells cultured in serum-free DMEM via *Ex-vivo* tissue explant culture. Meanwhile, testosterone had no effects on the osteogenic differentiation of AP cells.

**FIGURE 9:**
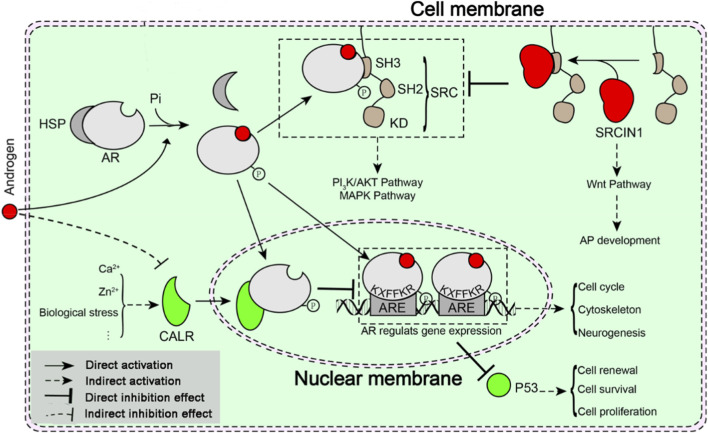
A schematic representation of possible androgen action in the initial development of pedicles. CALR regulates the transcriptional activity of AR to modulate gene expression. The AR modulates key effectors expression to regulate cell function, cell proliferation, cell cycle, cell survival, and neurogenesis. SRCIN1 suppresses the nongenomic signaling of AR. Genes and pathways modulated by AR are described in detail in the text.

There was strong evidence that androgen was a key factor in accelerating cell division of the AP cellular layer and triggering pedicle initiation (Figures 1, 2), which is consistent with the roles of androgen in regulating the pedicles development (Ba et al., 2022; Wang et al., 2025). It is known that the action of androgen is in target tissue via binding of the AR, which activates transcriptional program to regulate cell proliferation and differentiation. Previous studies show that CALR was one of the androgen-responsive genes and could be downregulated by castration at both the mRNA and protein levels (more than ten times) in the prostate (Alur et al., 2009; Wang, 1997; Zhu and Liu, 1997). However, in the present study with deer, expression level of CALR was upregulated after castration and downregulated when pre-castrated deer were treated with exogenous androgen treatment (which stimulates pedicle initiation). In addition, the expression of CALR was increased when the testosterone level decreased during the initial regeneration of antlers (Ba et al., 2019) and was negatively regulated by androgens, in contrast to the currently known model systems where all regulation is positive (Qianqian Guo et al., 2022). These studies also indicate that there should be an interaction between androgens and CALR in antler generation and regeneration. For example, CALR may bind to the conserved KXFFKR (where X is either G, A or V) sequence in the DNA-binding domain of AR and inhibit transcriptional activity of the AR to modulate gene expression and cell differentiation *in vivo* (Li et al., 1994). Therefore, downregulation of CALR might allow transcriptional activity of the AR to trigger pedicle initiation (Figure 9). However, the role of CALR in pedicle initiation is still unclear. Therefore, further work should focus on revealing the mechanisms involved in effects of CALR on pedicle initiation as well as determining the probable interacting genes and signaling pathways.

Beyond the indisputable importance of p53 as a tumor suppressor, p53 could also suppress the self-renewal of adult neural stem cells by negative regulation of cell proliferation and survival (Chang et al., 2006; Leskelä et al., 2006). A number of experiments show that androgen decreased cellular p53 levels and inhibited p53 protein accumulation in the nucleus to promote cell growth and survival (Figure 9) (Chang et al., 2006; Leskelä et al., 2006; Nantermet et al., 2004). In the present study, the protein expression level of p53 was also decreased significantly in the androgen-activated AP after injection of androgen. In this post-transcriptional mechanism, CDK5RAP3 could interact with CDKN2A/ARF and MDM2 and induce MDM2-dependent p53/TP53 ubiquitination, stabilization and activation in the nucleus, thereby promoting G1 cell cycle arrest and inhibition of cell proliferation (Chang et al., 2006; Leskelä et al., 2006). Consistent with above results, expression of CDK5RAP3 was upregulated in the undeveloped AP tissues, which probably promoted G1 cell cycle arrest and inhibition of cell proliferation. In addition, p53 and several cofactors genes and regulator genes have been positive selected in cervids comparing to tragulidae, antilocapridae, giraffidae, and bovidae (Ba et al., 2019), it is suggesting that p53 might be closely associated with antler development. Overall, androgens possibly triggered pedicle initiation by decreasing the expression of p53.

In the present study, SRCIN1 was 3.8-fold over-expressed in the androgen-activated AP tissues. Reported that over expression of SRCIN1 induced activation of the Wnt signaling pathway (Zhang et al., 2018). Hence it may be that SRCIN1 plays an important role in pedicle initiation by inducing activation of the Wnt signaling pathway (Figure 9). In this respect, the Wnt signaling pathway was found to play a role in the biology of AP development (Goss and Powel, 1985; Li and Suttie, 2012; Li and Suttie, 2001; Li et al., 2014). Over the past 2 decades evidence at the cellular and organismal level has implicated that androgens can bind to receptors in or around the plasma membrane, and activate cell-signaling pathways (Foradori et al., 2007; Migliaccio et al., 1996; Migliaccio et al., 1998). For instance, recent studies demonstrated that androgen-AR activated both PI3K/AKT and MAPK pathways by binding to the SH3 domain of Src (Migliaccio et al., 2000). However, SRCIN1 as a negative regulator of Src via binding to the Src SH3 domain directly suppressed activation of PI3K/AKT and MAPK pathways, and further inhibited cell proliferation (Damiano et al., 2010). Therefore, the AP cells may mainly be in a state of differentiation because of activation of Wnt pathway, rather than proliferation. Nevertheless, further study of the molecular regulatory mechanisms of SRCIN1 in the initial development of pedicles could be of interest.

The present study demonstrates that androgen supplementation during osteogenic induction significantly accelerates cell proliferation without enhancing matrix mineralization or altering the expression of key osteogenic transcription factors such as SP7 and SPARC. Strikingly, only BGN was markedly downregulated. Concordant with earlier reports, DHT or testosterone increased DNA synthesis and cell-cycle entry in osteoblast lineage cells (Vanderschueren et al., 2004). Wiren et al. (2008) found that AR over-expression stimulated proliferation yet suppressed mineral deposition. However, the present study shows that androgens promoted AP cell proliferation without affecting mineral deposition. This indicates that androgens did not activate the canonical BMP–RUNX2–SP7 pathway in the AP cells. BGN, a BMP2-inducible nucleator of hydroxyapatite, is likely suppressed indirectly via AR-mediated attenuation of BMP2 transcription (Maria Ceruti et al., 2021; Zara et al., 2011). This dissociation implies that Androgens maintain the AP cells in a highly proliferative state, ensuring the rapid growth of the pedicle during puberty.

## 5 Conclusion

This is the first comprehensive study of the proteomics for AP tissues involved in pedicle initiation underlying the regulation of androgens using 2D-DIGE. The results show that multiple pathways (e.g., cell cycle, gene expression and signal transduction) were involved in pedicle initiation under the influence of androgens. In particular, CALR, SRCIN1 and p53 may play an important role in pedicle initiation and antler regeneration. Testosterone stimulated pedicle initiation probably through promote cell proliferation instead of osteogenic differentiation of the AP cells. This research provides a valuable insight to explore the development of secondary sexual characters and to aid in the research of regenerative medicine and developmental biology.

## Data Availability

The data that support the findings of this study are available from the corresponding author upon reasonable request.
