# Supplementary material for: The regulation of pedicle initiation by androgens in sika deer (Cervus nippon)
Source: Front Cell Dev Biol. 2026 Mar 31;13:1708732. doi: 10.3389/fcell.2025.1708732 (PMC13076530; doi:10.3389/fcell.2025.1708732)
Supplement: Supplementary file 1 [file Supplementaryfile1.docx]

Fig. S1: Study design and workflow of 2D-DIGE analysis.

Fig. S2: Protein-protein interaction analysis between AR and the identified DEPs was performed using String (Version 10.5). Red: up-regulated proteins. Green: down-regulated protein. Yellow: proteins interacted with AR directly.

Fig. S3: Western blot analysis of p53, SMC4, SRCIN1, and GAPDH proteins in AP tissues from different groups.

Fig. S4: Western blot analysis of CALR proteins in AP tissues from different groups.
