# Supplementary figures and images for: The regulation of pedicle initiation by androgens in sika deer (Cervus nippon)
Source: Front Cell Dev Biol. 2026 Mar 31;13:1708732. doi: 10.3389/fcell.2025.1708732 (PMC13076530; doi:10.3389/fcell.2025.1708732)

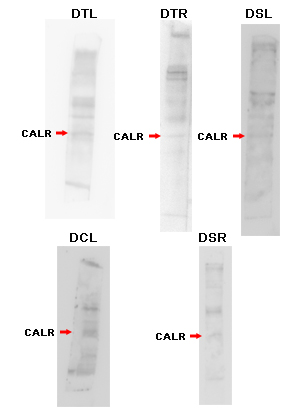

Supplement: Supplementary file 2 [file Image4.png]

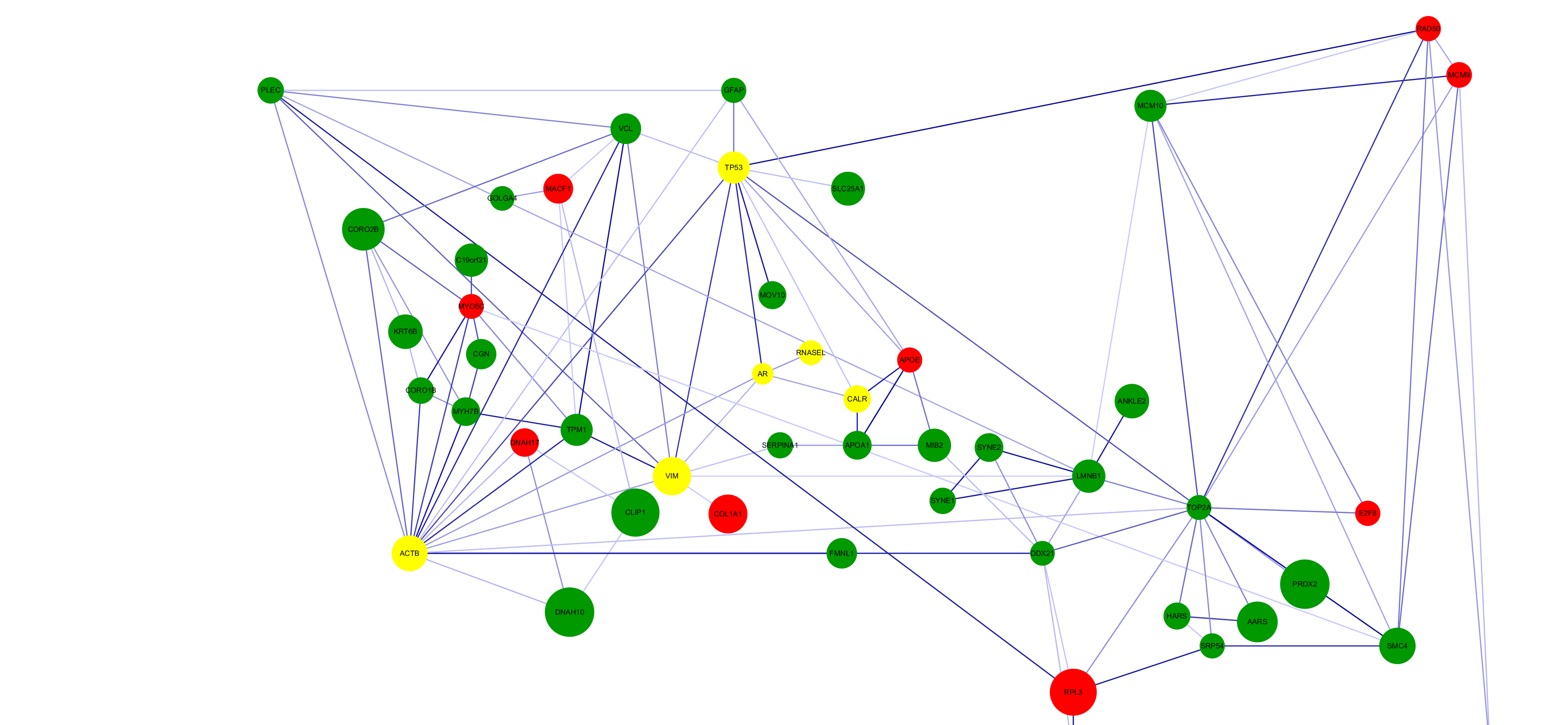

Supplement: Supplementary file 3 [file Image2.png]

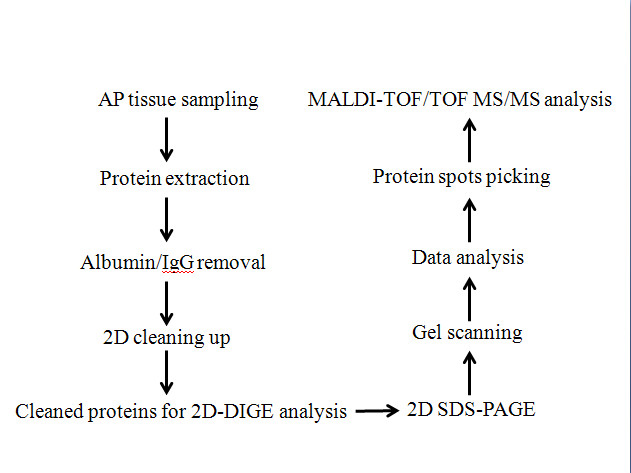

Supplement: Supplementary file 5 [file Image1.png]

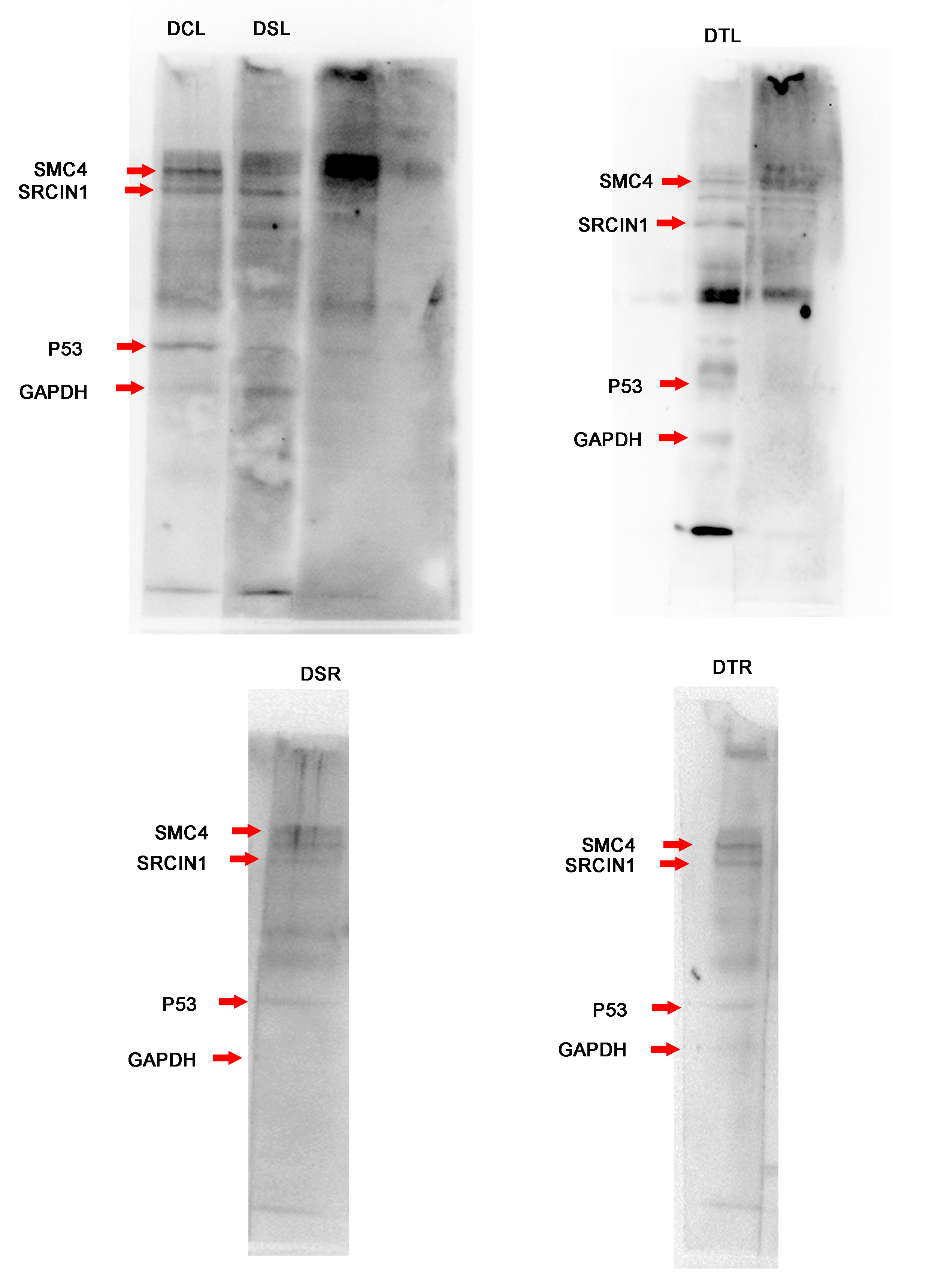

Supplement: Supplementary file 6 [file Image3.png]
